# Ectopic Functioning Adrenocortical Oncocytic Adenoma (Oncocytoma) with Myelolipoma Causing Virilization

**DOI:** 10.1155/2012/326418

**Published:** 2012-10-10

**Authors:** Lea F. Surrey, Ashesh A. Thaker, Paul J. Zhang, Giorgos Karakousis, Michael D. Feldman

**Affiliations:** ^1^Department of Pathology and Laboratory Medicine, The Hospital of the University of Pennsylvania, Philadelphia, PA 19104, USA; ^2^Department of Radiology, The Hospital of the University of Pennsylvania, Philadelphia, PA 19104, USA; ^3^Department of Surgery, The Hospital of the University of Pennsylvania, Philadelphia, PA 19104, USA

## Abstract

Functioning adrenal adenomas are well-described entities that can rarely occur outside the adrenal gland in the ectopic adrenal tissue. Similarly, myelolipoma is an another benign lesion of the adrenal tissue which can rarely occur outside the adrenal gland. We report the first case of a testosterone producing an extra-adrenal adrenocortical oncocytoma accompanied by a myelolipoma. The patient presented with virilization and elevated androgen levels. Imaging revealed a retroperitoneal mass, which histologically consisted of oncocytes and intermingled myelolipoma. Postoperative androgen levels decreased to normal. The tumor cells were strongly positive for inhibin and Melan-A, supporting the adrenal origin. This case demonstrates a diagnostic challenge in which correlation with histology, immunohistochemistry, and serum endocrine studies led to the final diagnosis.

## 1. Introduction

Adrenal cortical adenomas are common lesions of the adrenal cortex which may produce adrenal cortical hormones. These lesions are usually detected due to the clinical side effects of hormone production or found incidentally on imaging, especially in the case of nonfunctioning adenomas. Oncocytic adenomas in the adrenal gland are rare (approximately 132 reported cases) and have been described as adrenal oncocytomas [1]. Rarely adrenal cortical adenomas have been found outside the adrenal gland, in sites presumed to arise from ectopic adrenal tissue [2, 3]. However, adrenal oncocytoma has not been reported outside the adrenal gland. Myelolipoma is a benign proliferation of adipose tissue and trilineage hematopoietic elements typically found within the adrenal tissue. Myelolipomata have been associated with adrenal cortical adenomas both functioning and nonfunctioning but there are a few cases reported that occur outside the adrenal gland [4, 5]. We report a rare case of ectopic adrenocortical oncocytic adenoma (oncocytoma) accompanied by myelolipoma producing testosterone leading to virilization. To the best of our knowledge, there is no such case reported to date in the English literature.

## 2. Case Presentation

A 55-year-old female presented with hirsutism which included thinning scalp hair and increased hair over her back and arms. She had no other complaints and denied changes in weight, dizziness, urinary symptoms, changes in bowel habits, and skin changes. Her past medical history was notable for hypertension controlled with hydrochlorothiazide and ramipril as well as hypothyroidism controlled with levothyroxine. Her surgical history included two cesarean sections, partial thyroidectomy for a hyperplastic nodule, and cholecystectomy. Her examination was notable for hypertension, mild scalp alopecia, and increased hair growth on back and arms. The remainder of her examination, including pelvic exam, was unremarkable. Serum studies were notable for elevated total testosterone (635 ng/dL; normal 2–45 ng/dL), free testosterone (122.6 pg/mL; normal 0.1–6.4 pg/mL), and DHEA (268 mcg/dL; normal 15–170 mcg/dL). Aldosterone, cortisol, dopamine, epinephrine, norepinephrine, and creatinine were within normal limits. Renin was mildly elevated (4.9 ng/mL/hr; normal 0.5–4.0 ng/mL/hr). 

**Figure 1:**
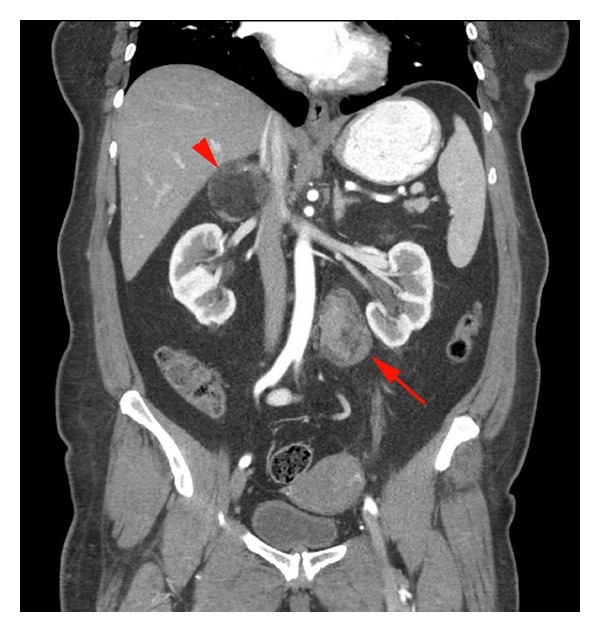
Coronal image from contrast enhanced CT of the abdomen/pelvis. A heterogeneous soft tissue mass is seen in the left para-aortic retroperitoneum, inferior to the renal hilum (arrow). A predominantly fat containing right adrenal mass is also noted with punctate calcifications (arrowhead). The left adrenal gland is normal.

The patient underwent pelvic ultrasound, which demonstrated normal ovaries; however, a retroperitoneal mass was incidentally discovered. Subsequently, computed tomography of the abdomen and pelvis was performed and the patient was found to have multiple abdominal lesions. Arising from the posteromedial upper pole of the left kidney was a 4.2 cm exophytic fatty lesion compatible with an angiomyolipoma. A predominantly fatty 5.5 cm right adrenal mass with punctate calcifications was also present and compatible with a myelolipoma. The third lesion was a heterogeneous, enhancing left retroperitoneal mass measuring nearly 7 cm and located just inferior to the left renal hilum (Figure 1). The patient underwent MRI to further characterize this mass, and it showed approximately 30% fatty and 70% nonfatty contents. Differential diagnosis based on imaging characteristics included retroperitoneal liposarcoma and primary retroperitoneal angiomyolipoma. Given the multiple fat-containing lesions, a diagnosis of tuberous sclerosis was entertained. Resection of the left retroperitoneal mass and renal mass was attempted; however, the renal lesion was not easily accessible at the time of surgery and only the retroperitoneal mass was removed.

**Figure 2:**
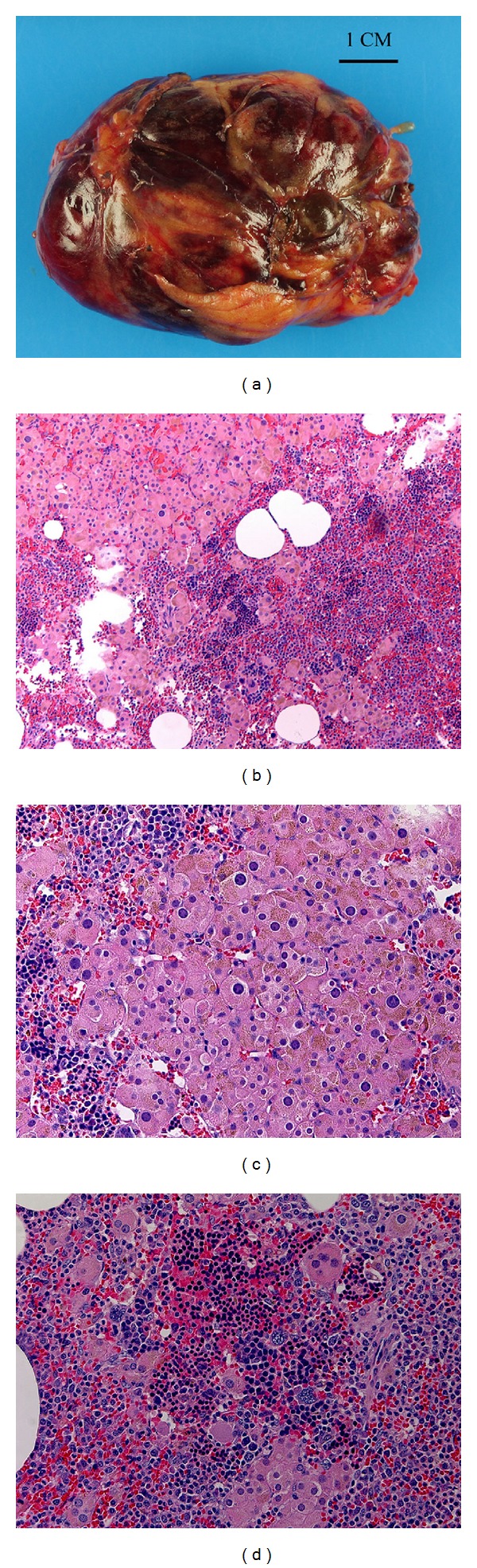
Gross examination of the retroperitoneal mass showed a red-tan smooth-surfaced lobulated mass with areas of fat (a). Microscopic examination showed oncocytes, adipocytes, and trilineage hematopoeisis intimately associated ((b), H&E, 100x). The oncocytes had abundant granular cytoplasm with lipofuscin. Random nuclear atypia is readily apparent but no mitotic activity was identified ((c), H&E, 200x). The oncocytes were interspersed with maturing erythroids and myeloid elements and megakaryocytes ((d), H&E, 200x).

**Figure 3:**
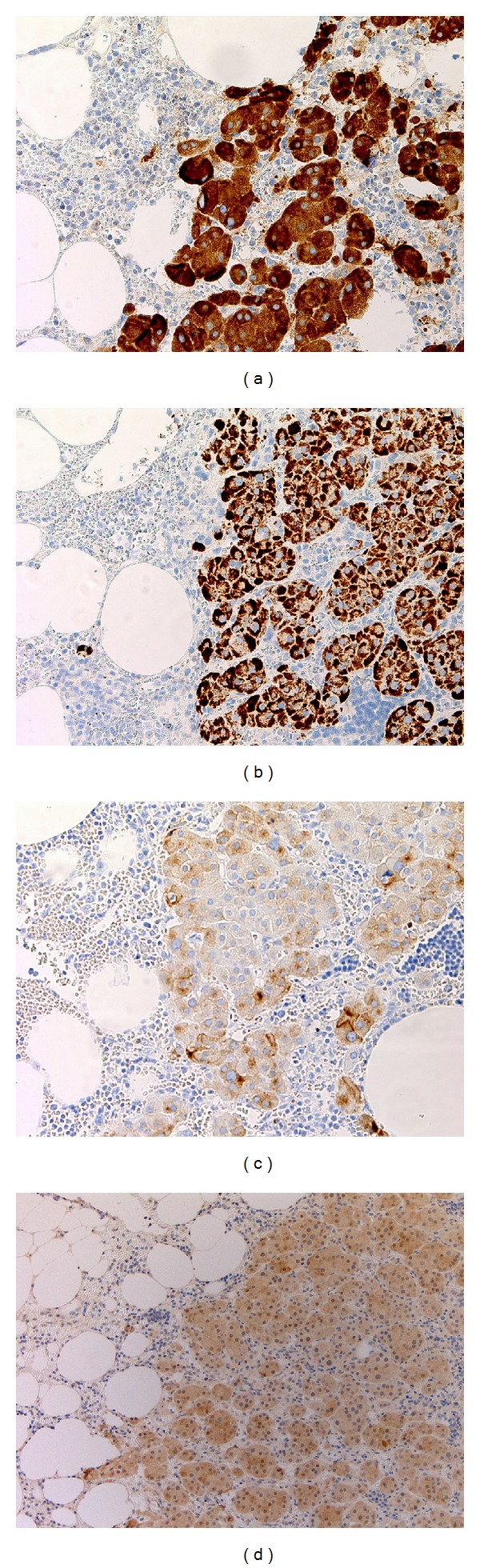
Immunohistochemical stains were strongly postive for Inhibin ((a), 200x) and Melan-A ((b), 200x) with weak positive staining for synaptophysin ((c), 200x) and calretinin ((d), 100x).

Grossly, the retroperitoneal excision consisted of a red-tan lobulated smooth-surfaced mass measuring 7.0 cm in the greatest dimension and weighing 55.5 grams (Figure 2(a)). The cut surface of the mass was brown to tan with areas of hemorrhage. Microscopic sections showed three intermingled components: adipose tissue, hematopoietic elements, and sheets or nests of polygonal cells with ample eosinophilic granular cytoplasm compatible with oncocytes (Figure 2(b)). The adipocytes were mature but showed some size variability. There were islands of hematopoietic tissue comprised of myeloid, erythroid, and megakaryocytic elements at different levels of maturation (Figure 2(d)). The oncocytes comprised the majority of the mass and had a rich capillary network. The oncocytes were also interspersed within the adipose and hematopoietic elements. Occasional yellow pigment consistent with lipofuscin was noted in some of the oncocytes. The nuclei of the oncocytes were round, some with prominent nucleoli, and random nuclear atypia with enlargement was evident (Figure 2(c)). No mitotic figures or necrosis was identified nor was there evidence of vascular invasion. The lesion did not appear to be microscopically encapsulated and the oncocytes extended to the margin of resection. The oncocytes showed strong immunoreactivity to inhibin and Melan-A, with weaker reactivity to calretinin, synaptophysin, and s100 (Figure 3). The oncocytes were negative for immunoreactivity to chromogranin, HMB45, epithelial membrane antigen, TTF-1, and thyroglobulin. Postoperative total testosterone decreased to within normal limits (5 ng/dL). On the basis of the pathologic and serologic information, the diagnosis of ectopic functioning adrenocortical oncocytic adenoma (oncocytoma) with myelolipoma was made.

## 3. Discussion

Oncocytomas are well-described entities composed of large cells with a granular eosinophilic cytoplasm containing many mitochondria. These neoplasms are known to arise in a variety of organs, including salivary glands, thyroid, parathyroid, kidney, and adenohypophysis. Also, a rare subset of adrenocortical adenomas with oncocytic features has been described and is frequently referred to as adrenocortical oncocytomas in the literature [1]. Supporting that our present case of an oncocytic neoplasm is of an adrenocortical origin is the strong immunohistochemical staining for inhibin and Melan-A. Adrenal cortex is an endocrine organ and is known to express some of the neuroendocrine markers such as synaptophysin and NSE; however, negative chromogranin staining in our case further supports the adrenal cortical origin rather than the adrenal medulla [6].

Given the adrenal cortical staining characteristics, we hypothesize that this retroperitoneal neoplasm most likely arose from ectopic adrenal tissue, which is most commonly found along the urogenital ridge structures, including the retroperitoneum, broad ovarian ligaments, mesentery, and appendix. Occasionally it has been reported that these ectopic adrenal rests can develop adrenocortical adenomas [7]. Most of the 132 described adrenocortical oncocytomas in the literature are nonfunctioning and are identified incidentally within the adrenal gland [1]. Virilization with adrenal oncocytoma has rarely been described [8]. In addition to oncocytic lesions within the adrenal gland, there are a few reports of retroperitoneal oncocytomas arising in apparent association with ectopic adrenal cortical tissue, as in our case [2, 3]. In both of these cases, these lesions were nonfunctioning, arose within the retroperitoneum, and were not associated with myelolipoma. Of interest, there has been one case report in the English literature (with a second report in the Chinese literature) of ovarian steroid tumor causing virilization in association with myelolipoma [9, 10]. Zhang and Lu noted that there was only rare ovarian stroma associated with the lesion and the remainder of the ovary and fallopian tube was unremarkable. Histologically and immunophenotypically the reported steroid cell tumor is very similar to the present case as well as the other reported cases of retroperitoneal oncocytoma, indicating that perhaps the origin of all of these lesions has a common precursor, namely, the ectopic adrenal tissue [9].

Myelolipomas are benign tumors comprised of mature fat cells and bone-marrow elements typically found in adrenal glands. They have been reported to occur in association with functioning and nonfunctioning cortical adenomas, congenital adrenal hyperplasia, and pheochromocytoma [5, 11–15]. The first case of myelolipoma involving a retroperitoneal ectopic adrenal tissue was reported in 1979 [4]. They can present a diagnostic challenge for radiologists as they may present very much like a well-differentiated liposarcoma [16]. To the best of our knowledge, in the English literature all reported cases of an extra-adrenal myelolipoma have not occurred in association with functioning (adenomatous) adrenal tissue. Since that time, no case of myelolipoma involving a functional extra-adrenal adenoma has been reported until this case. Thus, our case is unique in that it is a retroperitoneal ectopic adrenocortical oncocytic adenoma (alternatively, oncocytoma) producing testosterone that is also in association with myelolipoma.

Given that our patient in this case also has additional abdominal masses (right adrenal and left kidney), metastatic disease must be considered. Most reported cases of adrenal oncocytoma are benign but there have been malignant cases described [10, 17]. As with other oncocytic neoplasms, a definitive diagnosis of malignancy requires metastatic disease. While there is no established criteria for evaluating the malignant potential of extra-adrenal oncocytomas, the modified Weiss score for oncocytic adrenocortical neoplasms, developed in 2004, is currently the most widely accepted system for assessing oncocytic adrenal lesions [1, 18, 19]. Based on major (mitotic rate >5 per 50 HPF, atypical mitoses, venous invasion) and minor (size >10 cm, weight >200 grams, necrosis, capsular invasion, and sinusoidal invasion) criteria, a lesion is assigned as an adrenocortical oncocytoma (absence of all criteria), borderline neoplasm (any minor criteria), or oncocytic adrenocortical carcinoma (any major criteria) [1, 18, 19]. Our present case lacks any of the mentioned criteria and thus was classified as benign. The additional masses that were not sampled in this patient are also favored to be benign synchronous processes that are not hormonally functional, since the patient's testosterone has returned to normal. 

In summary, we report a case of a rare extra-adrenal adrenocortical oncocytoma, which is the first case reported to produce testosterone with the presentation of hirsutism and to be accompanied by myelolipoma, creating a diagnostic challenge. Correlation with histology, immunohistochemistry, and serum endocrine studies can aid in making the correct diagnosis.
